# The Neural Correlates of Shoulder Apprehension: A Functional MRI Study

**DOI:** 10.1371/journal.pone.0137387

**Published:** 2015-09-09

**Authors:** Hitoshi Shitara, Daisuke Shimoyama, Tsuyoshi Sasaki, Noritaka Hamano, Tsuyoshi Ichinose, Atsushi Yamamoto, Tsutomu Kobayashi, Toshihisa Osawa, Haku Iizuka, Takashi Hanakawa, Yoshito Tsushima, Kenji Takagishi

**Affiliations:** 1 Department of Orthopaedic Surgery, Gunma University Graduate School of Medicine, Maebashi, Gunma, Japan; 2 Department of Physical Therapy, Takasaki University of Health and Welfare, Takasaki, Gunma, Japan; 3 Department of Orthopaedic Surgery, National Hospital Organization Takasaki General Medical Center, Takasaki, Gunma, Japan; 4 Department of Advanced Neuroimaging, Integrative Brain Imaging Center, National Center of Neurology and Psychiatry, Kodaira, Tokyo, Japan; 5 Department of Diagnostic Radiology and Nuclear Medicine, Gunma University Graduate School of Medicine, Maebashi, Gunma, Japan; Wadsworth Center, UNITED STATES

## Abstract

Although shoulder apprehension is an established clinical finding and is important for the prevention of shoulder dislocation, how this subjective perception is evoked remains unclear. We elucidated the functional neuroplasticity associated with apprehension in patients with recurrent anterior shoulder instability (RSI) using functional magnetic resonance imaging (fMRI). Twelve healthy volunteers and 14 patients with right-sided RSI performed a motor imagery task and a passive shoulder motion task. Brain activity was compared between healthy participants and those with RSI and was correlated with the apprehension intensity reported by participants after each task. Compared to healthy volunteers, participants with RSI exhibited decreased brain activity in the motor network, but increased activity in the hippocampus and amygdala. During the passive motion task, participants with RSI exhibited decreased activity in the left premotor and primary motor/somatosensory areas. Furthermore, brain activity was correlated with apprehension intensity in the left amygdala and left thalamus during the motor imagery task (memory-induced), while a correlation between apprehension intensity and brain activity was found in the left prefrontal cortex during the passive motion task (instability-induced). Our findings provide insight into the pathophysiology of RSI by identifying its associated neural alterations. We elucidated that shoulder apprehension was induced by two different factors, namely instability and memory.

## Introduction

Dislocation of the glenohumeral (shoulder) joint is the most common dislocation in the human body [1] and is notorious for its high recurrence rate. The younger the patient when the initial dislocation occurs, the higher the recurrence rate [2]. Recent prospective studies showed that shoulder apprehension was a risk factor for the occurrence of glenohumeral instability [3, 4]. Moreover, shoulder apprehension obstructs daily life and sporting activities.

The apprehension test is used as an index of the physical state of the shoulder, wherein a positive result confirms anterior glenohumeral instability [5, 6]. Although shoulder apprehension is a clearly established clinical finding and is important for the prevention of first or recurrent shoulder dislocations, how this subjective perception is evoked remains unclear. One previous study examined the relationship between shoulder apprehension and neural adaptations in the central nervous system of patients with recurrent shoulder instability (RSI) [7], however the study did not assess brain activity associated with sensorimotor function. Moreover, the study subjects included individuals with both right- and left-sided RSI. This is problematic because of the different motor network patterns between the right and left shoulder joints. Therefore, the relationship between apprehension and shoulder motion in RSI has yet to be examined.

Evaluating the neural sources of apprehension in patients with RSI is crucial for understanding RSI pathophysiology, preventing first or recurrent dislocations after surgery, deciding whether surgery can improve the apprehension, and determining when patients can return to sporting activities. Here, using functional magnetic resonance imaging (fMRI), we tested our hypothesis that apprehension in patients with RSI would be induced by instability and by memory or imagery of the dislocation itself.

## Materials and Methods

### Subjects

Twelve healthy volunteers (4 women; mean age, 23.2 ± 3.2 years; age range, 20–29 years) and 14 patients with RSI (3 women; mean age, 28.2 ± 8.6 years; age range, 16–44 years) participated in this study. The selection criteria for patients with RSI included: 1) experienced a traumatic dislocation more than once, 2) a positive apprehension test [5] and a positive relocation test [8], and 3) experienced an isolated, right-sided RSI identified as a Bankart lesion by MR-arthrography, or arthroscopic assessment. Patients were excluded from the study if they had: 1) non-traumatic, multi-directional shoulder instability; 2) a history of a dislocation within a month prior to participation in the study; or 3) if the patient was less than 16 years of age. Brain activity was examined by fMRI. All participants were right-handed, with no history of neuropsychiatric disorders, other joint instability with apprehension, or contraindications for MRI. The Institutional Review Boards of Gunma University Hospital approved the study protocol. Participants were fully informed about the experimental procedures, and all provided written informed consent. For participants less than 18 years old, we obtained written informed consent from both the participants themselves and their parents.

### MRI acquisition

We used a 3-Tesla whole-body MRI scanner equipped with a circular polarization head coil (Siemens MAGNETOM Trio, A Tim System 3T; Erlangen, Germany). FMRI was obtained via an echo planar imaging sequence using the following parameters: whole brain, repetition time (TR) = 2500 ms, echo time (TE) = 30 ms, flip angle (FA) = 90°, 64 × 64 matrix, 38 slices, field of view (FOV) = 192 mm, voxel size 3 × 3 × 3 mm. For anatomical registration, T1-weighted three-dimensional structural images were also acquired with a magnetization-prepared, rapid-gradient echo sequence (TR = 2300 ms, TE = 3.26 ms, FA = 8°, 256 × 256 matrix, FOV = 256 mm, voxel size 1 × 1 × 1 mm).

### Experimental tasks

#### Motor imagery task

**Fig 1:**
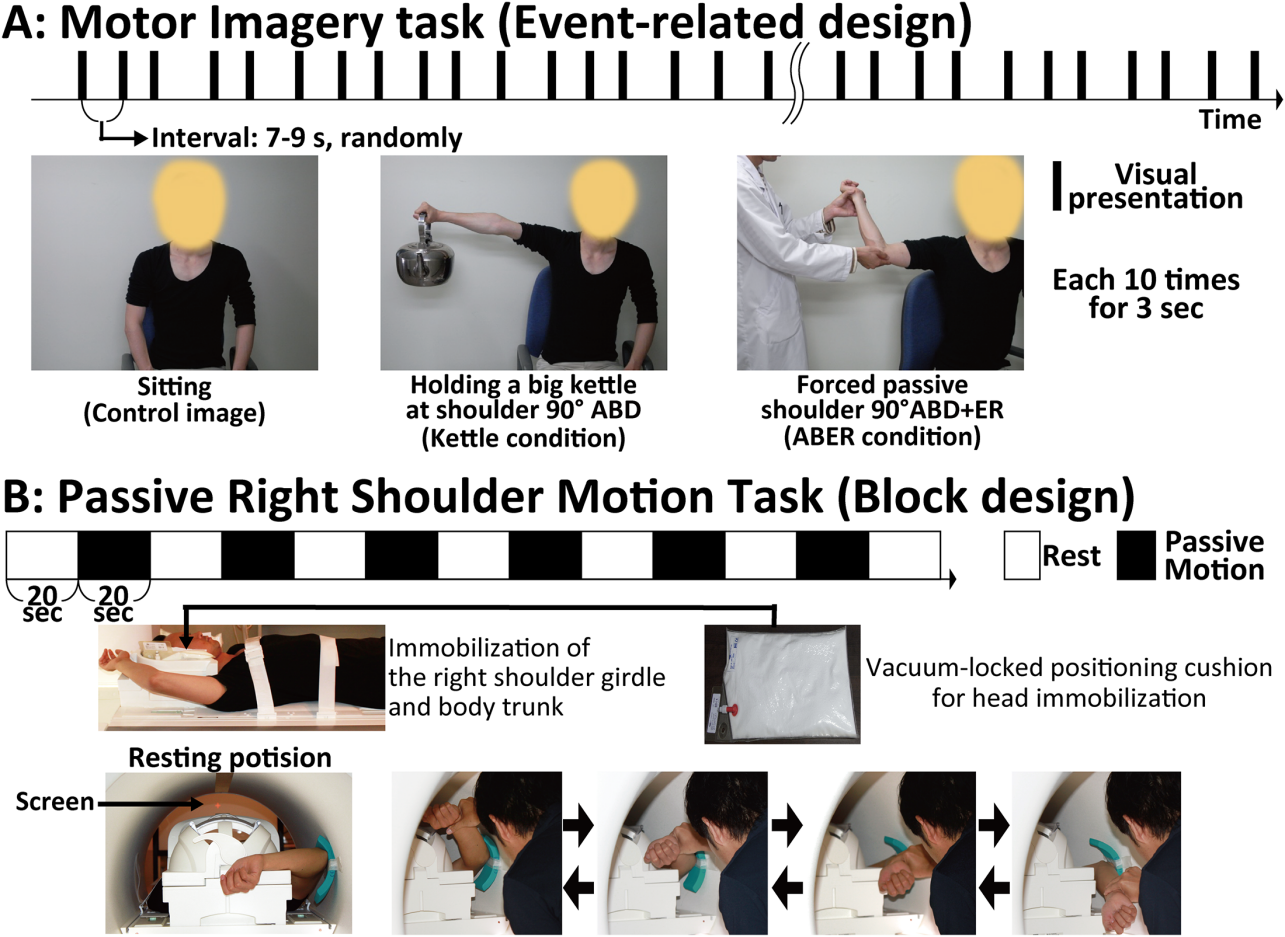
Descriptions of the motor imagery and passive motion tasks. **A**: Motor imagery task during fMRI. The pictures presented were as follows: a man sitting down without moving his shoulder (as a control image of non-shoulder motion), a man holding a big kettle at 90° of shoulder abduction, and a man performing a forced passive shoulder external rotation at 90° of shoulder abduction. These three pictures were presented randomly for 3 s every 7–9 s, and each picture was presented 10 times. **B**: Passive right shoulder motion task during fMRI. The right shoulder girdle and body trunk were tightly fixed to the scanner bed with non-elastic bandages. Then, form pads and vacuum cushions were used to minimize head motion. In the MRI scanner, participants’ right shoulders were passively rotated in an external and internal motion with approximately 90° abduction at approximately 1 Hz by an experienced orthopaedic surgeon. The participants’ shoulders were moved in response to cues projected on a screen located on the foot side of the participants. The passive shoulder motion and resting conditions were alternated every 20 s.

To detect brain activity associated with imagery- and memory-induced apprehension, three pictures were presented randomly for 3 s (presentation interval, 7–9 s; each picture was presented 10 times) in an fMRI scanner. The pictures were of a man sitting down without moving his shoulder (non-shoulder motion [control] condition), a man holding a big kettle with his right shoulder abducted to 90° (kettle condition), and a man performing a forced passive external shoulder rotation with his right shoulder abducted to 90° (ABER condition). We instructed participants to imagine that they were performing the movement in the presented picture (Fig 1A). Image timing was controlled by Presentation software (Neurobehavioral systems, Albany, CA, USA), and after the task performance, participants reported their perceived shoulder apprehension intensity in each condition using a 0–10 Numeric Rating Scale (NRS: 0 [no apprehension]–10 [worst apprehension imaginable]).

#### Passive right shoulder motion task

To minimize head motion during scanning, the right shoulder girdle and body trunk were tightly fixed to the scanner bed with non-elastic bandages. To further minimize motion, form pads and vacuum cushions (Vac-Lock Cushion, CIVCO, Coralville, IA, USA) were used (Fig 1B).

To detect brain activity associated with motion-induced shoulder apprehension, participants’ right shoulders were passively rotated in an external and internal motion with approximately 90° abduction (like the position in the shoulder apprehension test) at approximately 1 Hz by an experienced orthopaedic surgeon in an MRI scanner. The cues in the passive motion task were controlled by projecting pictures on a screen located on the foot side of the participants. The passive shoulder motion and resting conditions were alternated every 20 s (Fig 1B). After performing this task, we asked participants to rate their average shoulder apprehension intensity using the NRS.

### fMRI data analysis

Imaging data were preprocessed using SPM8 (Wellcome Department of Imaging Neuroscience, UCL, London, UK) and implemented in MATLAB (MathWorks, Natick, MA, USA). The first four volumes in each experimental run were discarded to allow for T1 equilibrium effects, and the remaining functional images were corrected for differences in slice acquisition timing. The motion-corrected images were then spatially normalized to fit the Montreal Neurological Institute (MNI) template based on the standard stereotaxic coordinate system. Subsequently, all images were smoothed with an isotropic full-width at half maximum Gaussian kernel of 8 mm.

Statistical analysis was performed using SPM8, and a vector representing event onset was modelled as the main regressor for the first-level general linear model. This analysis was performed for each participant to test the correlation between fMRI signal changes and a train of delta functions convolved with the canonical hemodynamic response function and its temporal derivative. Six parameters (three rotational and three translational) representing head motion were included in the design matrix as covariates of no interest, and global signal normalization was only performed between runs. Low-frequency noise was removed using a 128-s high-pass filter and serial correlations were adjusted using an auto-regression model. We computed summary images reflecting the effects of interest on fMRI signals by applying linear contrasts to the parameter estimates. A second-level random-effect group analysis was then performed to identify voxels that showed a significant difference in activity between the motor imagery (kettle condition and/or ABER condition) and control conditions in each group and between the passive motion condition and rest. In all comparisons, the threshold was initially set at a voxel-wise height level of *P* < 0.05 corrected for multiple comparisons (family-wise error; FWE). The cytoarchitectonic definition of significant brain activity was identified according to the SPM8 anatomy toolbox, where applicable.

### Between-groups fMRI analysis

The two subject groups were compared directly via a second-level random-effects analysis. Areas of differential activity were detected using a between-groups analysis, with the following contrasts: patients with RSI > controls and controls > patients with RSI. The thresholds were set to *P* < 0.05 corrected for the between-groups analysis and *P* < 0.001 uncorrected for multiple comparisons.

### Association between brain activity and apprehension

In order to understand the alterations in brain activity associated with shoulder apprehension in patients with RSI during each task, we used a general linear model (GLM) to test the correlation between brain activity and apprehension intensity. As a measure of apprehension intensity, we used the results obtained from the NRS as a covariate within the RSI group, since the control participants in this study had never felt shoulder apprehension. Furthermore, Pearson’s product-moment correlations were calculated using IBM SPSS Statistics 22 software program (IBM Japan, Ltd, Tokyo, Japan) to verify that correlations found between the beta values extracted in instances of significant brain activation and apprehension intensity were significant.

## Results

### Controlling for confounding effects of differences between groups and head motion during both tasks

We first ensured that our findings were not likely to be substantially influenced by confounding effects from age and gender differences between groups or head motion artefacts. There were no significant differences in age (P = 0.08) or gender (P = 0.50) between the patient and control groups. To assess head motion during the tasks, the functional images were first visually inspected for quality to ensure they did not contain sources of extreme noise such as image distortion and signal drop. Then, the estimated translations (x, y and z) and rotations (pitch, roll and yaw) were extracted from the realignment parameters for each subject. In the motor imagery task, the mean estimated head motions in the x, y, z, pitch, row, and yaw directions were minimal: 0.11±0.14 mm, 0.03±0.27 mm, 0.04±0.21 mm, 0.001±0.007°, -0.001±0.004°, and -0.001±0.003°, respectively. In the passive shoulder motion task, the mean estimated head motions in the x, y, z, pitch, row, and yaw directions were likewise minimal: 0.18±0.08 mm, 0.08±0.07 mm, 0.13±0.08 mm, 0.002±0.001°, 0.002±0.001°, and 0.002±0.001°, respectively. No subjects exhibited extreme motion (>4 mm translation, >5 degrees rotation)[9] in either task.

### Motor imagery task (Fig 2)

**Fig 2:**
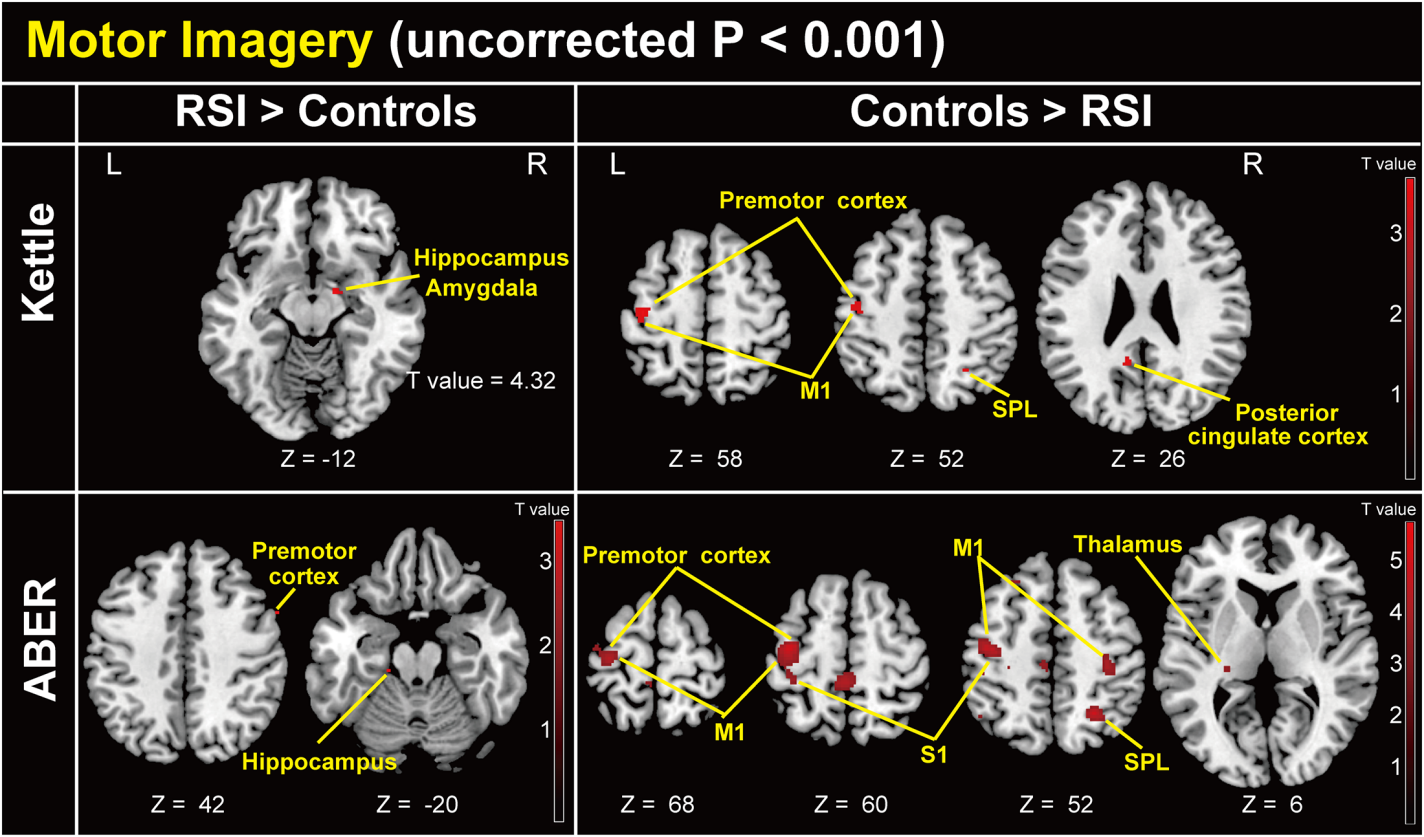
Group-level statistical parametric map showing the categorical comparison of hemodynamic changes in the motor imagery task (whole-brain uncorrected *P* < 0.001). **Upper left**: Comparison of patients with RSI > controls in the kettle condition relative to the control condition. **Upper right**: Comparison of controls > patients with RSI in the kettle condition relative to the control condition. **Lower left**: Comparison of patients with RSI > controls in the ABER condition relative to the control condition. **Lower right**: Comparison of controls > patients with RSI in the ABER condition relative to the control condition. z (mm): z-coordinate in Montreal Neurological Institute (MNI) space, which is the basic brain template from the MNI. Plus and minus values indicate the dorsal and ventral directions, respectively. Red bar indicates a T value. M1: primary motor cortex, S1: primary somatosensory cortex, SPL: superior parietal lobule.

Significant differences were found in several contrasts between patients with RSI and controls. Patients with RSI showed significantly higher brain activity in the right hippocampus and amygdala in the kettle minus control condition (Table 1A), and in the right precentral gyrus and left hippocampus in the ABER minus control condition (Table 1B). Healthy participants showed significantly higher activity in several brain regions in the kettle relative to the control condition (Table 1C) and the ABER relative to the control condition (Table 1D). Interestingly, in the controls > patients with RSI comparison, brain activity in the kettle condition relative to the control condition was similar to that of the ABER condition relative to the control condition.

**Table 1:** Results of group-level statistical parametric mapping with specific contrasts in the motor imagery task.

Activity clusters (functional anatomy)	Coordinates (mm)			T values
	x	y	z	
**Kettle condition—control condition in motor imagery**				
**(A) RSI patients > controls (uncorrected *P* < 0.001)**				
Right hippocampus, amygdala	14	-6	-12	4.32
**(B) Controls > RSI patients**				
Left precentral gyrus (PMC, M1)	-40	-18	58	3.93
Left postcentral gyrus (S1)	-42	-16	50	3.71
Left posterior cingulate cortex	-10	-50	28	3.91
Right rectal gyrus	16	24	-12	3.89
Right superior occipital gyrus	20	-98	22	3.78
Right inferior parietal lobule	26	-56	52	3.69
Right superior parietal lobule	36	-64	62	3.61
Right rectal gyrus	4	38	-18	3.59
Right middle orbital gyrus	6	40	-14	3.57
Left paracentral lobule	-8	-38	66	3.48
**ABER condition—control condition in motor imagery**				
**(C) RSI patients > controls (uncorrected *P* < 0.001)**				
Right precentral gyrus	58	10	42	3.65
Left hippocampus	-18	-28	-20	3.59
**(D) Controls > RSI patients (uncorrected *P* < 0.001)**				
Left precentral gyrus (PMC)	-36	-14	56	5.88
Left postcentral gyrus (S1)	-46	-30	48	4.05
Right inferior parietal lobule (SPL)	26	-56	52	5.09
Left paracentral lobule	-4	-38	64	4.96
Right inferior temporal gyrus	62	-24	-22	4.33
Right precentral gyrus (M1)	34	-26	52	4.23
Left inferior parietal lobule (IPC)	-46	-58	56	4.07
Left thalamus	-24	-26	4	3.95
Left fusiform gyrus	-28	-68	-10	3.91
Left middle frontal gyrus	-24	30	54	3.83
Right fusiform gyrus	38	-42	-24	3.82

IPC: Inferior parietal cortex, M1: Primary motor cortex, PMC: Premotor cortex, S1: Primary somatosensory cortex, SPL: Superior parietal lobule. A: The kettle condition minus the control condition (uncorrected, *P* < 0.001) in the patients with RSI > controls comparisons. B: The kettle condition minus the control condition in the controls > patients with RSI comparisons (uncorrected, *P* < 0.001). C: The ABER condition minus the control condition in the patients with RSI > controls comparisons (uncorrected, *P* < 0.001). D: The ABER condition minus the control condition in the controls > patients with RSI comparisons (uncorrected, *P* < 0.001). x, y, z (mm): coordinates in Montreal Neurological Institute (MNI) space. x-coordinate: Plus and minus values indicate the direction of right and left, respectively. y-coordinate: Plus and minus values indicate the anterior and posterior directions, respectively. z-coordinate: Plus and minus values indicate the dorsal and ventral directions, respectively.

### Passive shoulder motion task

**Fig 3:**
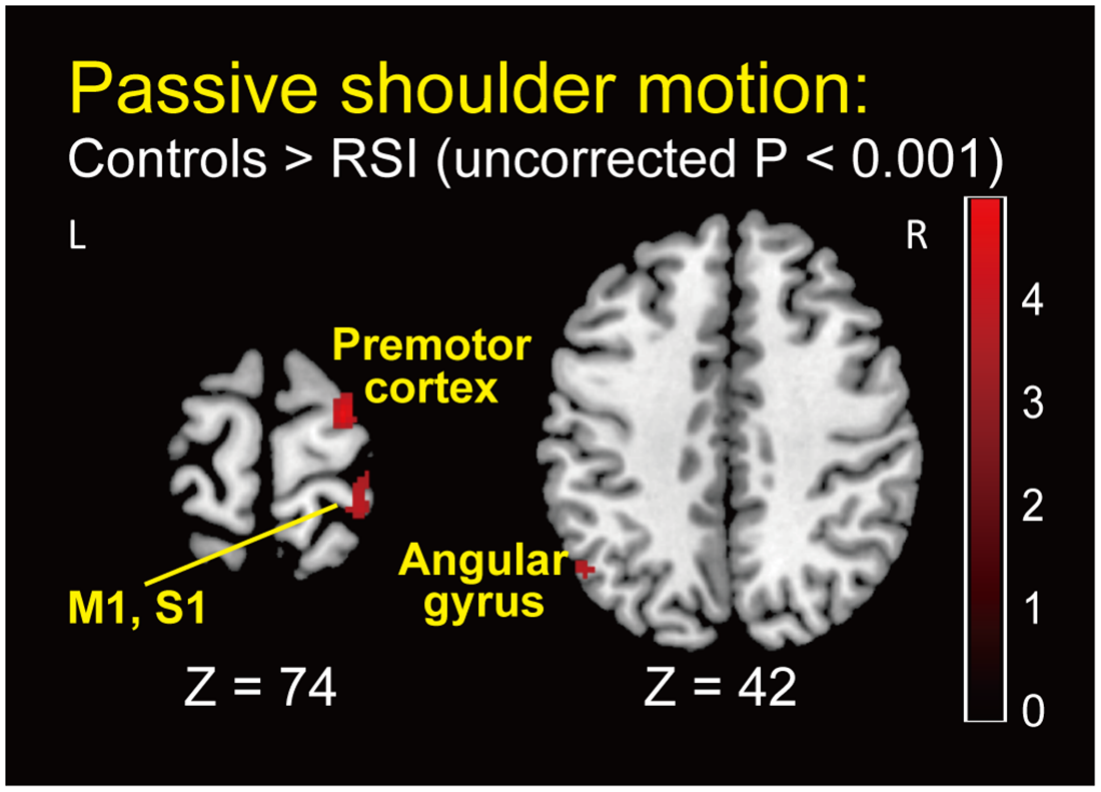
Group-level statistical parametric map showing the categorical comparison of hemodynamic changes in the comparison of controls > patients with RSI in the passive shoulder motion task (whole-brain uncorrected *P* < 0.001). z (mm): z-coordinate in Montreal Neurological Institute (MNI) space, which is the basic brain template from the MNI. Plus and minus values indicate the dorsal and ventral directions, respectively. Red bar indicates T value. M1: primary motor cortex, S1: primary somatosensory cortex.

Compared with controls, no significant brain activity occurred in patients with RSI. Conversely, in the controls > patients with RSI comparison, brain activity was significantly elevated in the right pre- and postcentral gyrus, right superior frontal gyrus, right medial temporal pole, left middle and inferior temporal gyri, and left angular gyrus (Fig 3 and Table 2).

**Table 2:** Results of group-level statistical parametric mapping with specific contrasts in the passive shoulder motion task.

Activity clusters (functional anatomy)	Coordinates (mm)			T values
	x	y	z	
Passive shoulder motion: Controls > RSI patients (uncorrected *P* < 0.001)				
Right superior frontal gyrus (PMC)	24	-10	74	4.86
Right medial temporal pole	38	6	-26	4.24
Left inferior temporal gyrus	-54	-56	-4	3.80
Right postcentral gyrus (S1)	30	-42	74	3.37
Right precentral gyrus (PMC, M1)	32	-30	74	3.80
Left angular gyrus (IPC)	-48	-62	42	3.72
Left middle temporal gyrus	-62	-32	-6	3.55

IPC: Inferior parietal cortex, M1: Primary motor cortex, PMC: Premotor cortex, S1: Primary somatosensory cortex. x, y, z (mm): coordinates in Montreal Neurological Institute (MNI) space. x-coordinate: Plus and minus values indicate the direction of right and left, respectively. y-coordinate: Plus and minus values indicate the anterior and posterior directions, respectively. z-coordinate: Plus and minus values indicate the dorsal and ventral directions, respectively.

### Correlation between apprehension and brain activity

When participants were viewing the control condition, there was no shoulder apprehension observed in either group (NRS = 0). When participants imagined the kettle condition, the mean shoulder apprehension intensity using the NRS was 1.9 (range, 0–7) in patients with RSI and 0 in controls. In the ABER condition, this value was 7.6 (range, 5–10) in patients with RSI and 0 in controls. Covariate analysis using the NRS for shoulder apprehension intensity showed that in the ABER condition, patients with RSI exhibited significantly elevated brain activity in the anterior cingulate cortex (ACC), right S1, hippocampus, parahippocampal gyrus, middle occipital gyrus, superior parietal lobule, lingual gyrus, bilateral cerebellum, left thalamus, fusiform gyrus, precuneus, and calcarine gyrus during the motor imaginary. In the kettle condition, significantly elevated brain activity was found in the left amygdala, thalamus, inferior frontal gyrus, right rolandic operculum, and middle occipital, lingual, and rectal gyri during the motor imaginary.

**Fig 4:**
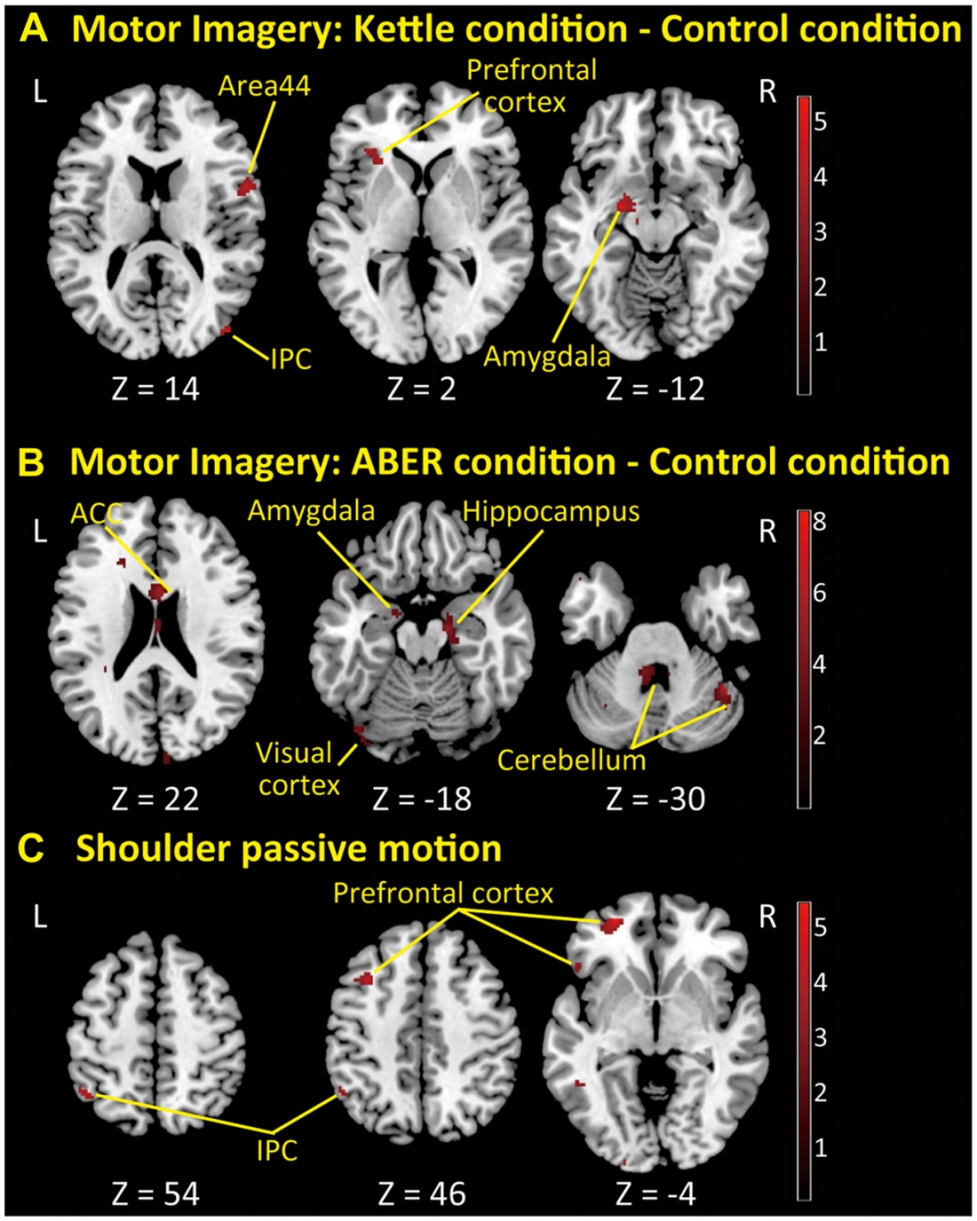
Correlation analyses between shoulder apprehension intensity and brain activity using tasks and motion as covariates. **A**: Correlation analysis between apprehension intensity and brain activity using covariates showing the categorical comparison of hemodynamic changes between the kettle and control conditions (uncorrected *P* < 0.005 for display purposes). **B**: Correlation analysis between apprehension intensity and brain activity using covariates showing the categorical comparison of hemodynamic changes between the ABER and control conditions (uncorrected *P* < 0.005 for display purposes). **C**: Correlation analysis between apprehension intensity and brain activity using covariates showing the categorical comparison of hemodynamic changes between passive shoulder motion and baseline (uncorrected *P* < 0.005 for display purposes). z (mm): z-coordinate in Montreal Neurological Institute (MNI) space, which is the basic brain template from the MNI. Plus and minus values indicate the dorsal and ventral directions, respectively. Red bar indicates T value. ACC: Anterior cingulate cortex, IPC: Inferior parietal cortex.

When the participants’ shoulders were passively rotated externally and internally at a shoulder abduction of 90°, the mean shoulder apprehension intensity on the NRS was 5.9 (range, 0–10) in patients with RSI and 0 in controls. In the passive shoulder motion task, significantly elevated activity was found in the left inferior parietal lobule and in the left middle temporal, superior orbital, and middle frontal gyri (Fig 4 and Table 3). Pearson’s product-moment correlation analysis revealed a strong positive correlation (Pearson’s correlation coefficient > 0.75, *P* < 0.05) in either area between activity in all of these areas and apprehension intensity.

**Table 3:** Results of group-level statistical parametric mapping with apprehension-intensity as a covariate and correlation analysis.

Activity clusters (functional anatomy)	Coordinates (mm)				Correlation Analysis	
	x	y	z	T values	*r* values	*P* values
**(A) Kettle—control in motor imagery (uncorrected *P* < 0.001)**						
Left amygdala	-22	-6	-12	5.36	.830	.0002
Right Rolandic operculum (BA 44)	58	8	10	4.81	.800	.0005
Right middle occipital gyrus (IPC)	46	-84	14	4.67	.798	.0006
Right lingual gyrus (BA 18)	14	-84	-6	4.22	.773	.0011
Right rectal gyrus	4	20	-18	4.12	.779	.0010
Left thalamus	-12	-16	-8	4.07	.764	.0015
Left inferior frontal gyrus	-32	30	2	3.96	.753	.0019
**(B) ABER—control in motor imagery (uncorrected *P* < 0.001)**						
Right hippocampus	14	-2	-28	8.14	.880	< .0001
Right cerebellum	44	-52	-30	6.59	.831	.0002
Right anterior cingulate cortex	2	10	22	5.35	.823	.0003
Right hippocampus	16	-14	-18	5.35	.832	.0002
Left thalamus	-28	-30	-2	5.02	.806	.0005
Left fusiform gyrus	-40	-80	-16	4.75	.799	.0006
Right superior parietal lobule (SPL)	24	-58	70	4.48	.797	.0007
Left cerebellum	-2	-46	-26	4.42	.809	.0005
Right middle occipital gyrus	24	-88	4	4.38	.798	.0006
Right parahippocampal gyrus (Hippocampus)	24	-22	-26	4.34	.775	.0011
Left thalamus	-2	-14	12	4.21	.773	.0012
Left precuneus	-22	-48	10	4.15	.768	.0013
Left calcarine gyrus (BA 17)	0	-102	2	4.10	.766	.0014
Right primary somatosensory cortex	30	-22	40	4.07	.762	.0015
**(C) Shoulder passive motion task (uncorrected *P* < 0.001)**						
Left middle temporal gyrus	-64	-30	-10	5.60	.831	.0004
Left superior orbital gyrus	-26	54	-4	5.59	.830	.0004
Left middle frontal gyrus	-34	18	46	4.44	.806	.0009
Left inferior parietal lobule (IPC)	-44	-56	54	4.29	.798	.0011

BA: Brodmann area, IPC: Inferior parietal cortex, SPL: Superior parietal lobule. *r* values: Pearson’s correlation coefficient. A: In the kettle condition minus the control condition (uncorrected, *P* < 0.001). B: In the ABER condition minus the control condition (uncorrected, *P* < 0.001). C: In the passive shoulder motion task (uncorrected, *P* < 0.001). x, y, z (mm): coordinates in Montreal Neurological Institute (MNI) space. x-coordinate: Plus and minus values indicate the direction of right and left, respectively. y-coordinate: Plus and minus values indicate the anterior and posterior directions, respectively. z-coordinate: Plus and minus values indicate the dorsal and ventral directions, respectively.

## Discussion

Here, we measured brain activity in patients with RSI and healthy participants during passive shoulder motions and shoulder motor imagery tasks. Compared to controls, patients with RSI exhibited elevated brain activity in the hippocampus during motor imagery, a significant decrease in brain activity normally induced by motor imagery associated with shoulder motion, and elevated activity during passive shoulder motion in the left premotor cortex relative to the left premotor cortex, left primary motor and somatosensory cortices (M1 and S1, respectively), and right cerebellum in the control group. Additionally, significant correlations were found between apprehension ratings and brain activity associated with affect, fear, anxiety, and motor control during motor imagery tasks, and the brain activity correlated with apprehension intensity induced by motor imagery was completely different from that induced by passive shoulder movement.

### Motor imagery task

In the patients with RSI vs. controls comparison, we found significantly elevated brain activity in the hippocampus and amygdala during the kettle condition and in the prefrontal cortex and hippocampus during ABER imagery. Several lines of evidence support the notion that the amygdala is responsible for detecting, generating, and maintaining fear-related emotions. Specifically, the amygdala is implicated in the recognition of fearful facial expressions [10], feelings of fear after procaine induction [11], fear conditioning [12–14], and evoking fearful emotional responses from direct stimulation [15]. The amygdala is also important for detecting environmental threats [16, 17] and for coordinating responses to threat and danger [18, 19]. Several human imaging studies support the role of the medial prefrontal cortex, including the ACC and amygdala, in extinction learning of discrete conditioned stimuli [20, 21]. Likewise, the amygdala, ventromedial prefrontal cortex, and hippocampus are implicated in extinction recall [22, 23]. Here, the involvement of these brain regions suggests that motor imagery associated with shoulder motion not only evoked unpleasant or fearful memories involved in the prior experience of shoulder dislocation, but were also judged as threatening. Furthermore, the motor imagery task used here might have induced fear extinction. In the controls > patients with RSI comparison, we found significantly higher brain activity in the superior parietal lobule and motor network ipsilateral to the affected shoulder, and in the premotor cortex, M1, S1, and thalamus contralateral to the affected shoulder. Previous studies have identified the brain areas activated in equal proportions in movement versus imagery tasks, and those areas that are not [24]. The perceived vividness of motor imagery was parametrically associated with brain activity within sensorimotor areas [25]. These studies support our results, as participants without RSI were able to imagine shoulder motion with more precision, vividness, and/or strength than were patients with RSI. In other words, picturing shoulder motion induced normal patterns of activity and was more successful in control participants than in patients with RSI. This finding suggests that RSI might affect shoulder motion imagery, which is important for precise shoulder motor control. Thus, RSI might be a neurophysiological dysfunction rather than a simple peripheral musculoskeletal injury.

### Passive shoulder motion task

Although patients with RSI experienced shoulder apprehension, no significant brain activity occurred in patients with RSI compared with controls. This might be due to the variability of brain activity related to shoulder apprehension between patients. Alternatively, brain activity related to shoulder apprehension could be masked or might have overlapped with motion-induced brain activity since proprioceptive afferents could be strongly activated during passive shoulder motion. However, compared to patients with RSI, control subjects showed significantly elevated activity in the right pre- and postcentral gyrus, right superior frontal gyrus, right medial temporal pole, left middle and inferior temporal gyrus, and left angular gyrus. A previous positron emission tomography study showed that attributing intentions to others was associated with complex activity involving the right middle and medial prefrontal cortex including Brodmann’s area 9, the right medial temporal pole, and the bilateral middle temporal gyri [26]. This might indicate that our healthy participants attended to the examiner rather than to their own shoulder movement during the passive motion task. In other words, patients with RSI attended to their own shoulder movement because of apprehension or shoulder instability. The angular gyrus is involved in several spatial cognition tasks including the spatial analysis of external sensory information and internal mental representations [27]. This evidence supports the idea that patients with RSI have a decreased ability for spatial integration and cognition. It has been shown that the right pre- and postcentral gyrus, including the ipsilateral M1 and S1, are normally activated by motor imagery and motor execution tasks [24]. This suggests that patients with RSI have decreased motor imagery abilities and/or reduced proprioceptive afferents from the injured shoulder.

### Brain activity correlated with apprehension intensity

Shoulder apprehension induces specific reorganization in the functional connectivity of apprehension-related areas, including the primary somatosensory motor areas, dorsolateral prefrontal cortex, dorsal anterior cingulate cortex/dorsomedial prefrontal cortex, and anterior insula [7]. Our results obtained from the motor imagery task are partially consistent with this notion; however, we also detected brain activity in the amygdala and hippocampus. The difference between the studies might be a result of the variation in visual stimulation; specifically, our pictures might have been more effective at inducing apprehension. Here, we showed that brain activity in the prefrontal cortex, a region involved in the cognitive control of motor behaviour, and the inferior parietal cortex, involved in repetitive passive motion [28] were correlated with shoulder apprehension. However, we did not detect activity in the amygdala or hippocampus during the passive shoulder motion task. It is possible that there was a slight increase in brain activity due to dislocation anxiety since the subject may have already felt shoulder apprehension just by being placed in the starting position in the scanner. Interestingly, the brain activity that correlated with apprehension intensity induced by motor imagery was completely different from that induced by passive shoulder motion. As described previously, brain activity in the medial prefrontal cortex, including the ACC, amygdala, and hippocampus, which we observed in apprehension intensity induced by motor imagery, is associated with the extinction of discrete conditioned stimuli [20, 21] or extinction recall [22, 23]. Thus, these studies suggest that memory- or imagery-induced shoulder apprehension differs from actual movement-induced shoulder apprehension. This result might be useful in interpreting residual post-operative shoulder apprehension and may help identify the need for additional therapies for treating memory-induced shoulder apprehension. In the future, new treatment strategies that aim to reduce activity of the amygdala and/or hippocampus should be considered.

### Limitations

Several limitations should be considered about ways to induce apprehension. First, participants’ shoulders during the passive shoulder motion task could not be completely positioned at 90° abduction, like the position in the shoulder apprehension test, because of restricted space in the MRI scanner. Therefore, it is possible that we did not induce enough shoulder apprehension, like that induced in clinical examinations. However, we think the task did induce some level of apprehension because we could detect apprehension-related brain activity following passive shoulder motion. Second, we considered the evaluation of apprehension induced by active shoulder motion, but we did not perform this task because of the (1) risk of shoulder dislocation during the task, (2) difficulty of matching the motion performance between controls and patients with RSI, (3) restricted space to evoke apprehension by active shoulder motion in the scanner, and (4) effect of motion artefacts, which would worsen the data quality because of head motion occurring along with active shoulder motion. Thus, we decided not to perform the active shoulder motion task related to apprehension because patients’ safety was our first priority.

## Conclusion

Here, we show brain activity associated with both shoulder instability itself and dislocation apprehension experienced by patients with RSI. Using fMRI to evaluate shoulder apprehension in patients with RSI might help to preoperatively distinguish memory-induced apprehension, which should not be treated with surgery, from instability-induced apprehension, which should be treated with surgery. Regarding treatment strategies for RSI, this represents an important translational study; therefore, we will collect additional data in the future and apply our findings in clinical settings.

## Supporting Information

S1 Fig Group-level statistical parametric map using age as a covariate in the motor imagery task and passive shoulder motion task. (TIF)

S2 Fig Coverage of MRI acquisition. (TIF)

S3 Fig Brain activation in the passive shoulder motion task. (TIF)

S4 Fig Averaged group-level statistical parametric map after random resampling to fit same sample size in the motor imagery task and passive shoulder motion task. (TIF)

S5 Fig Group-level statistical parametric map showing the categorical comparison of hemodynamic changes in the contrast of controls > patients with RSI in both the kettle and ABER conditions relative to the control condition in the motor imagery task (whole-brain FWE-corrected *P* < 0.05). (TIF)

S1 Movie Movie of the passive shoulder motion task. (MP4)

S1 Table Mean estimated head motions in each trial. (XLSX)

S1 Text Age difference between groups. (DOCX)

S2 Text Head motion estimates during the shoulder passive motion task. (DOCX)

S3 Text Confidence estimates with a random equal size sample of healthy vs. RSI. (DOCX)

S4 Text The categorical comparison of hemodynamic changes in both the kettle and ABER conditions relative to the control condition in the motor imagery task. (DOCX)
