# Supplementary figures and images for: The Neural Correlates of Shoulder Apprehension: A Functional MRI Study
Source: PLoS One. 2015 Sep 9;10(9):e0137387. doi: 10.1371/journal.pone.0137387 (PMC4564220; doi:10.1371/journal.pone.0137387)

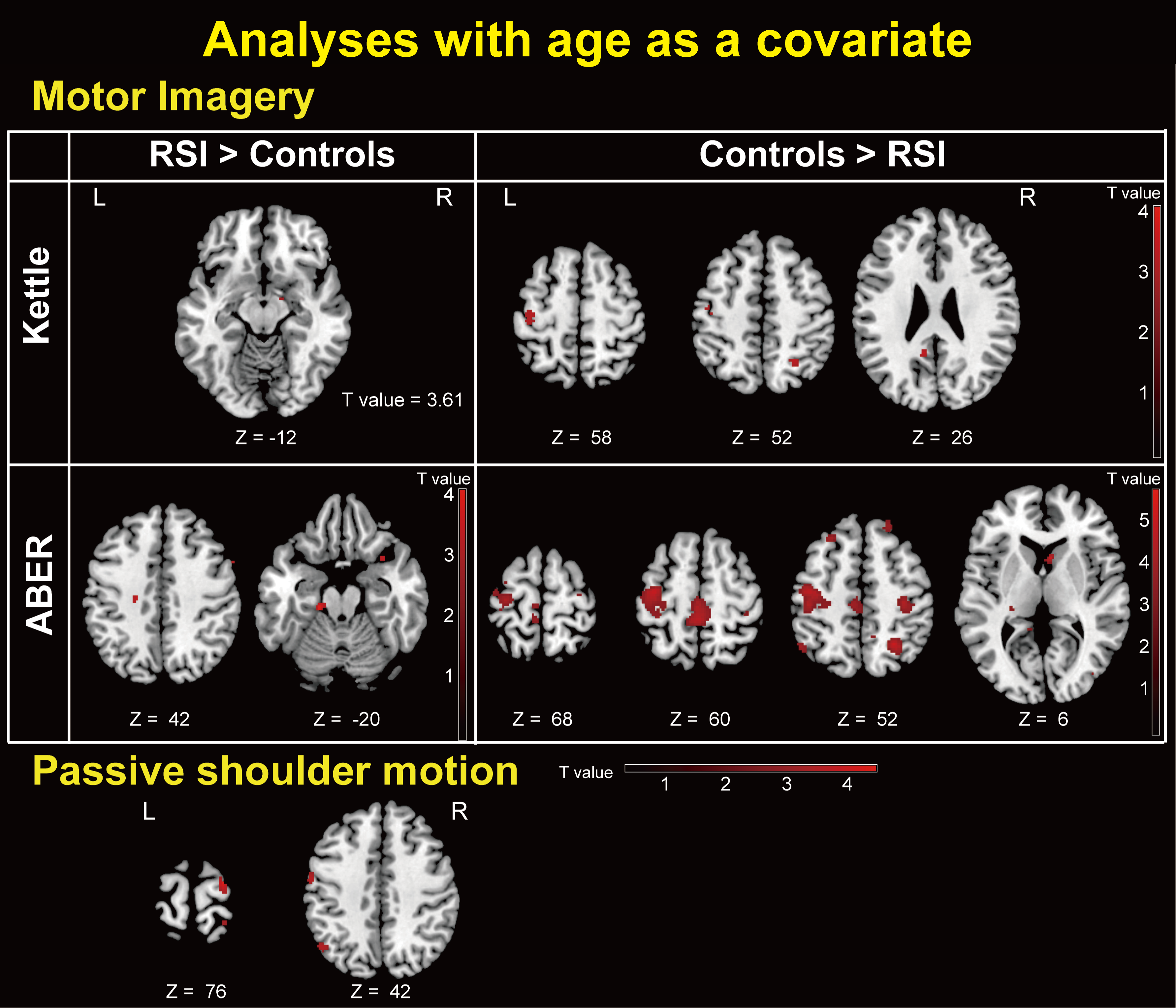

Supplement: S1 Fig — (TIF) [file pone.0137387.s001.tif]

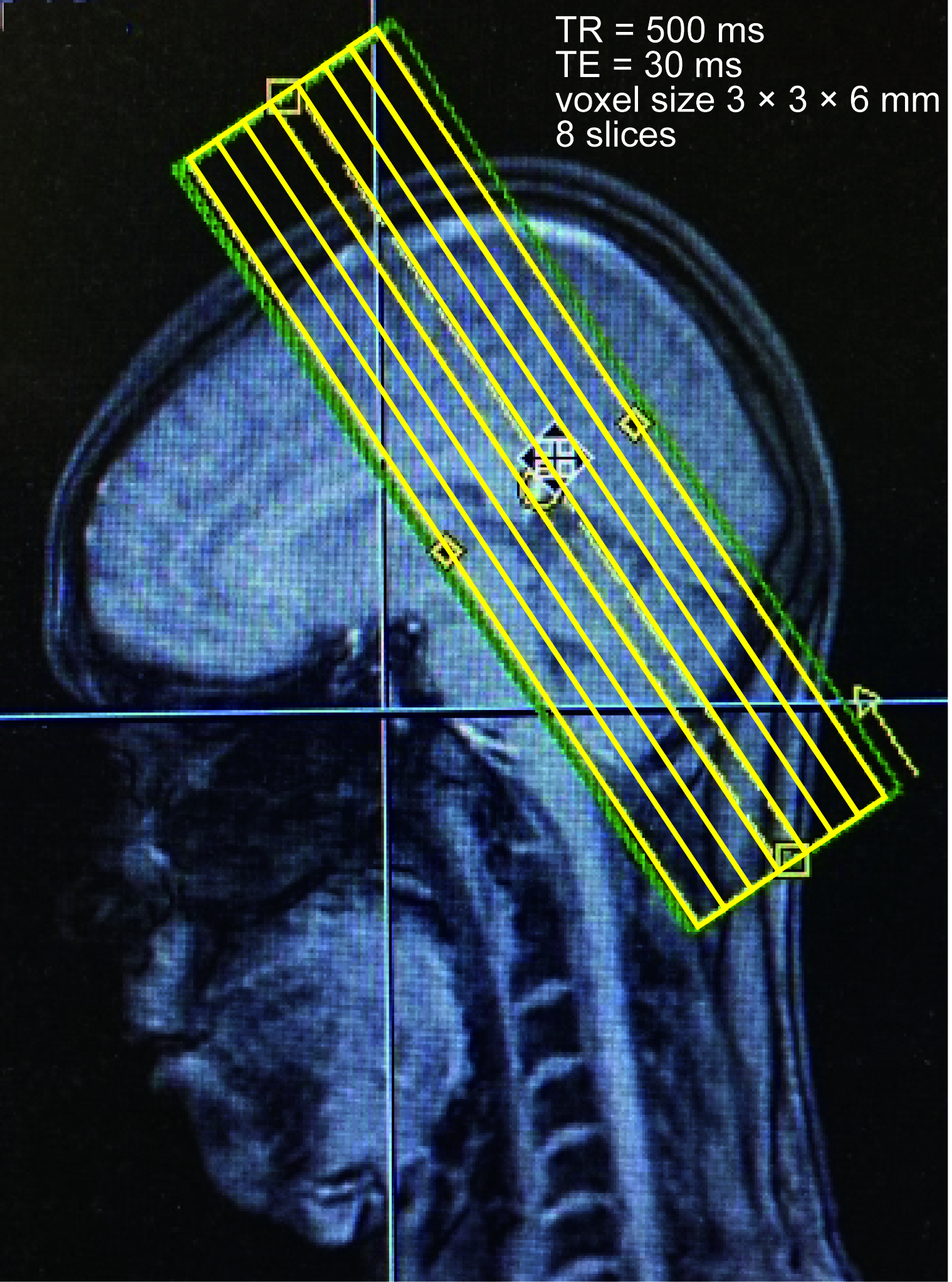

Supplement: S2 Fig — (TIF) [file pone.0137387.s002.tif]

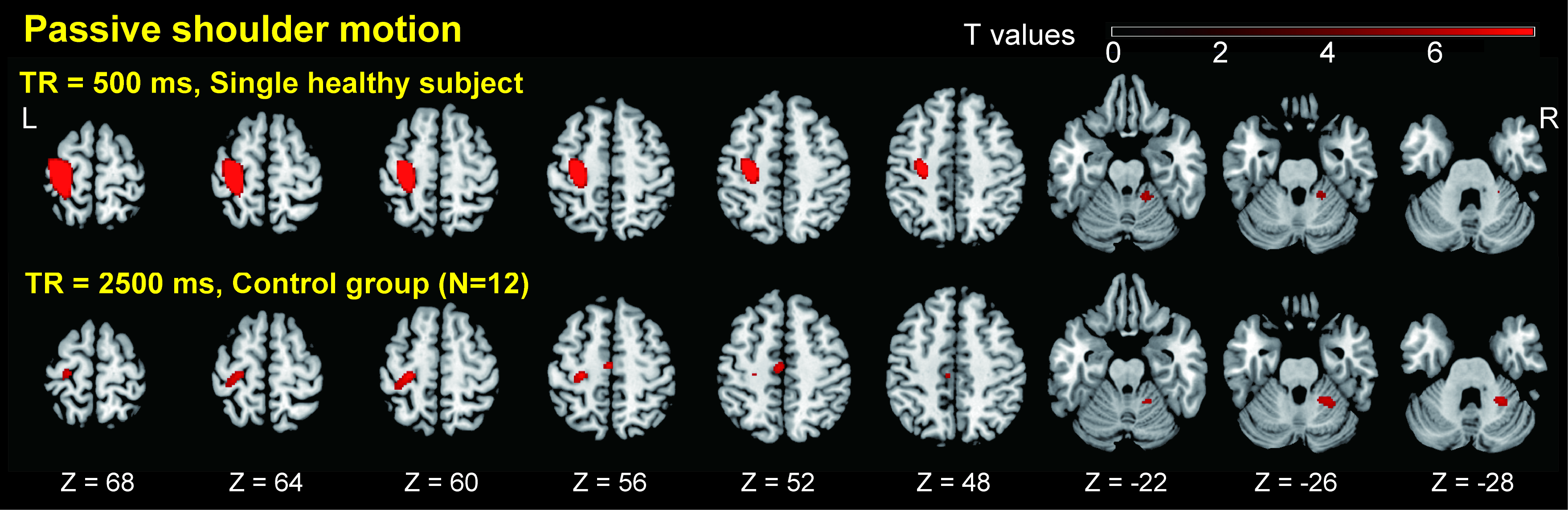

Supplement: S3 Fig — (TIF) [file pone.0137387.s003.tif]

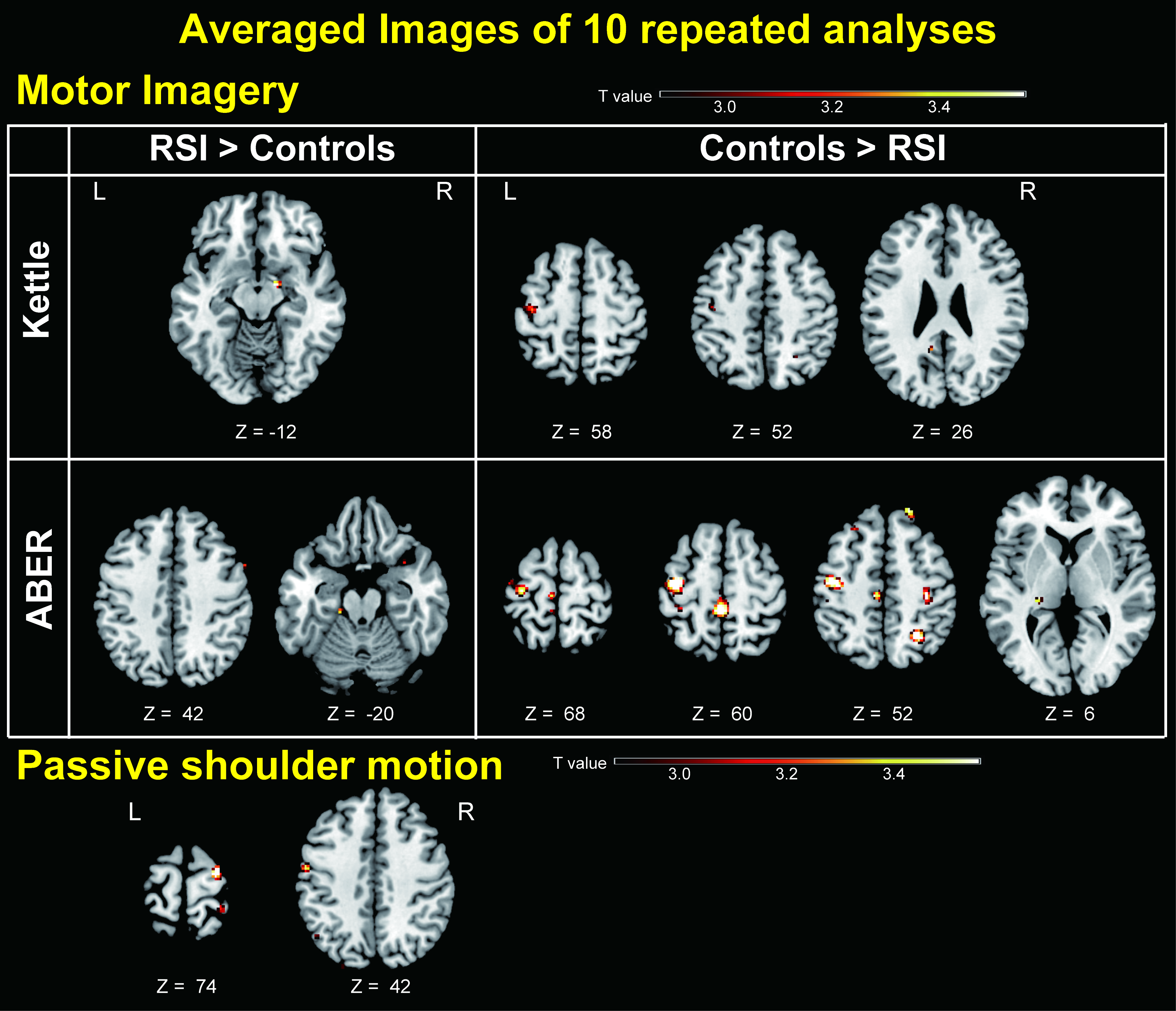

Supplement: S4 Fig — (TIF) [file pone.0137387.s004.tif]

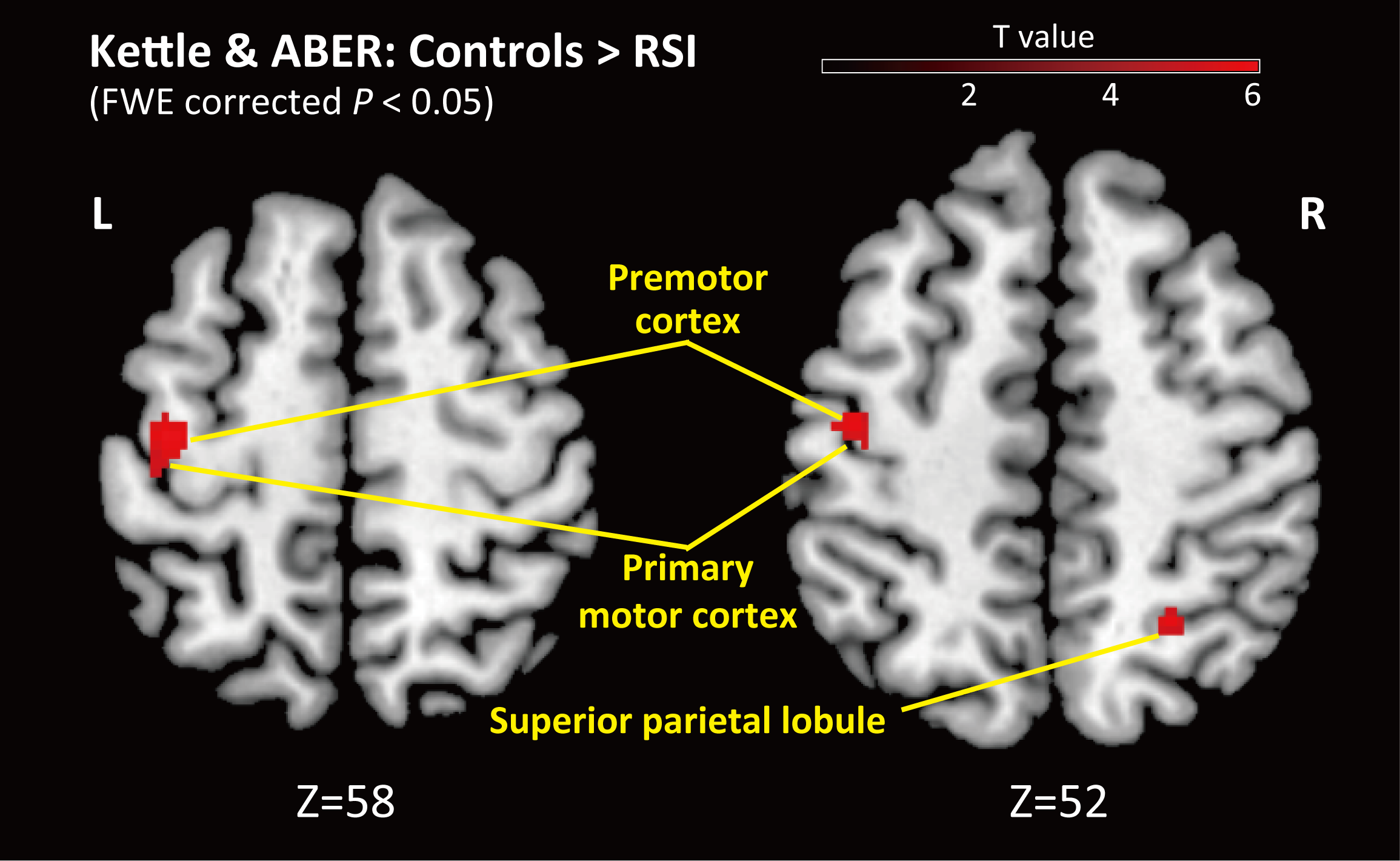

Supplement: S5 Fig — (TIF) [file pone.0137387.s005.tif]
