# Supplementary material for: The Neural Correlates of Shoulder Apprehension: A Functional MRI Study
Source: PLoS One. 2015 Sep 9;10(9):e0137387. doi: 10.1371/journal.pone.0137387 (PMC4564220; doi:10.1371/journal.pone.0137387)
Supplement: S1 Text — (DOCX) [file pone.0137387.s008.docx]

**Age difference between groups**

Although there was no significant difference in the age between patients and controls (P=0.08), the P–value for age difference was near our cut off for statistical significance. Therefore, we did additional analysis using age as a covariate to reduce the effects of the age difference. These results were similar to those obtained without covariate analysis. Therefore, we believe that the effects of the age difference were minor (S1 Fig.).
