# Supplementary material for: The Neural Correlates of Shoulder Apprehension: A Functional MRI Study
Source: PLoS One. 2015 Sep 9;10(9):e0137387. doi: 10.1371/journal.pone.0137387 (PMC4564220; doi:10.1371/journal.pone.0137387)
Supplement: S2 Text — (DOCX) [file pone.0137387.s009.docx]

**Head motion estimates during the shoulder passive motion task**

To inspect the head motion estimates during the shoulder passive motion task in detail, we performed the shoulder passive motion task, which comprised 6 trials, lasting 20 s, in a healthy participant using a short TR. MRI was conducted with the echo planar imaging sequence using the following parameters: whole brain, repetition time (TR) = 500 ms, echo time (TE) = 30 ms, flip angle (FA) = 90°, 64 × 64 matrix, 8 slices, field of view (FOV) = 192 mm, voxel size 3 × 3 × 6 mm. Bilateral cortical motor and somatosensory areas, and cerebellar lobes were covered with an oblique coronal acquisition (S2 Fig). The mean head motion estimates in x, y, z, pitch, row, and yaw directions were -0.24±0.06 mm, 0.12±0.04 mm, 0.01±0.08 mm, -0.0006±0.0005°, 0.0049°±0.0010°,and 0.0012±0.0005°, respectively (S2 Table). We believe that these head motion values are within the allowable range for treating head motion artifacts as nuisance covariates.

Brain activity was significantly elevated in the left pre- and postcentral gyrus (including M1 and S1), left supramarginal gyrus (including secondary somatosensory cortex (S2)), right superior temporal gyrus (including S2), and right cerebellum (S3 Fig). This pattern of activation was largely consistent with the results obtained for healthy participants in the control group. Taken together, these results indicate that the effects of head motion on brain activation data in the passive shoulder motion task were minor.
