# Supplementary material for: The Neural Correlates of Shoulder Apprehension: A Functional MRI Study
Source: PLoS One. 2015 Sep 9;10(9):e0137387. doi: 10.1371/journal.pone.0137387 (PMC4564220; doi:10.1371/journal.pone.0137387)
Supplement: S3 Text — (DOCX) [file pone.0137387.s010.docx]

**Confidence estimates with a random equal size sample of healthy vs. RSI volunteers**

To confirm that our analysis of the control versus RSI groups was not biased by differences in group size, we randomly selected 10 out of the 12 healthy volunteers and 10 out of the 14 patients with RSI using Excel RAND function (Microsoft Excel 2013^®^), which generates a random real number. We repeated this selection 10 times and the results of the analysis for each selection were averaged. The results for both the motor imagery and passive shoulder motion tasks were consistent with those obtained by comparing 12 healthy volunteers with 14 patients with RSI (S4 Fig.).
