# Supplementary material for: The Neural Correlates of Shoulder Apprehension: A Functional MRI Study
Source: PLoS One. 2015 Sep 9;10(9):e0137387. doi: 10.1371/journal.pone.0137387 (PMC4564220; doi:10.1371/journal.pone.0137387)
Supplement: S4 Text — (DOCX) [file pone.0137387.s011.docx]

**The categorical comparison of hemodynamic changes in the contrast of controls > patients with RSI in both the kettle and ABER conditions relative to the control condition in the motor imagery task**

In the controls > patients with RSI comparison, brain activity in the kettle condition relative to the control condition was similar to that of the ABER condition relative to the control condition. Thus, we pooled these two shoulder motor imageries together, which led to the detection of an elevation in brain activity in the left precentral gyrus (premotor cortex, T value=6.15), the left paracentral lobule (T value=5.85), and the right inferior parietal lobule (superior parietal lobule, T value=5.97) in the controls > patients with RSI comparison (FWE, *P* < 0.05) (S5 Fig.).
